# Supplementary material for: Establishing a mass spectrometry-based system for rapid detection of SARS-CoV-2 in large clinical sample cohorts
Source: Nat Commun. 2020 Dec 3;11:6201. doi: 10.1038/s41467-020-19925-0 (PMC7713649; doi:10.1038/s41467-020-19925-0)
Supplement: Supplementary file 1 — Supplementary Information [file 41467_2020_19925_MOESM1_ESM.pdf]

Supplementary Information for:

Establishing a mass spectrometry-based system for rapid detection of SARS-CoV-2 in large clinical sample cohorts

Cardozo K.H.C., Lebkuchen A., Okai G.G., Schuch R.A., Viana L.G., Olive A.N., Lazari C.S., Fraga A.M., Granato C.F.H., Pintão M.C.T, Carvalho V.M.

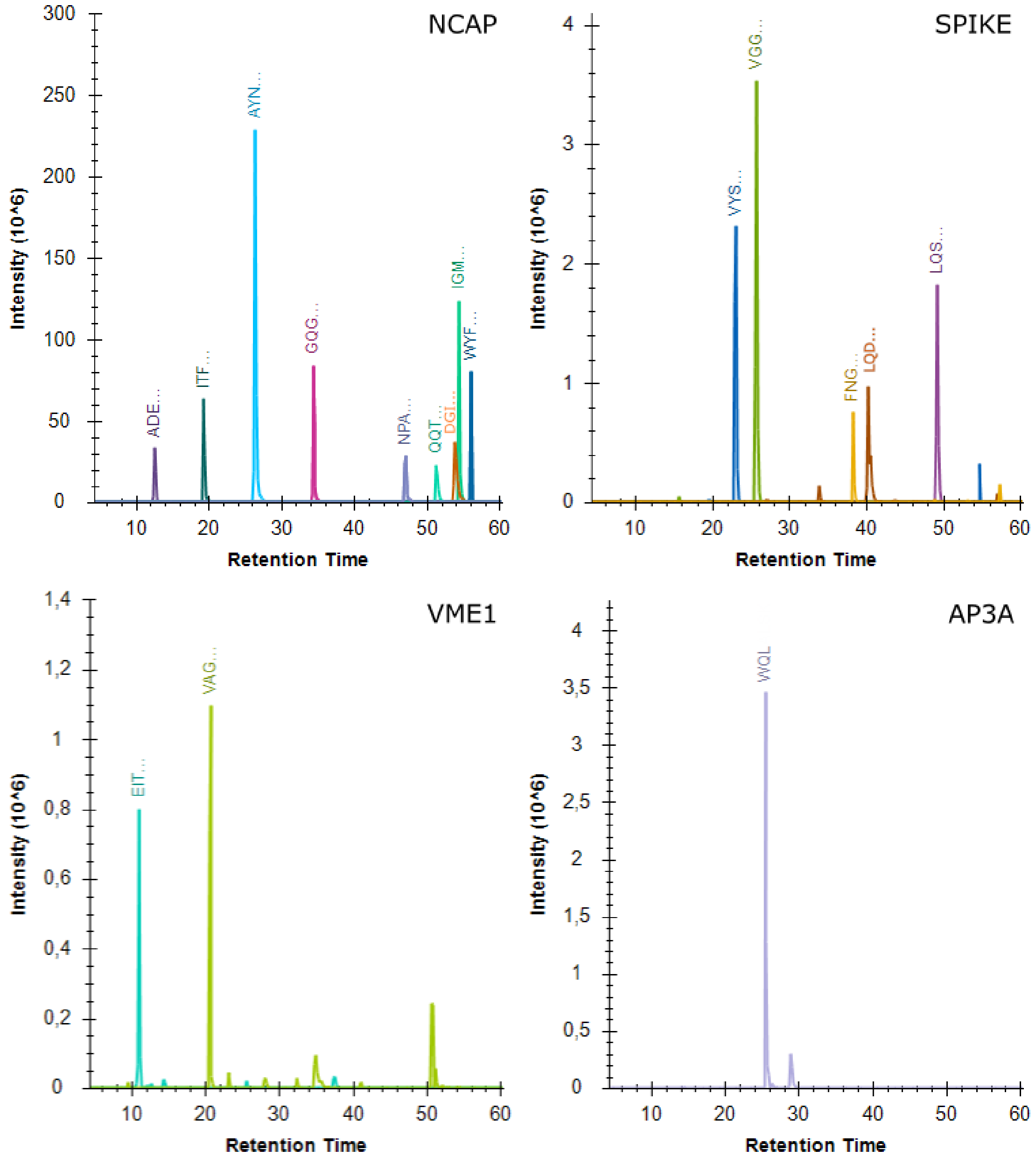

Supplementary figure 1. Sixty minutes parallel reaction monitoring (PRM) chromatograms of SARS-CoV-2-positive respiratory tract samples showing the most promising peptides for nucleoprotein (NCAP), spike glycoprotein (SPIKE), membrane protein (VME1), and protein 3a (AP3A). The first three residues of each peptide are used to label peptide peaks. NCAP (nucleoprotein): ADE (ADETQALPQR), ITF (ITFGGPSDSTGSNQNGER), AYN (AYNVTQAFGR), GQG (GQGVPINTNSSPDDQIGYYR), NPA (NPANNAAIVLQLPQGTTLPK), QQT (QQTVTLLPAADLDDFSK), DGI (DGIIWVATEGALNTPK), IGM (IGMEVTPSGTWLTYTGAIK), WYF (WYFYYLGTGPEAGLPYGANK). SPIKE (spike glycoprotein): VYS (VYSTGSNVFQTR), VGG (VGGNYNYLYR.), FNG (FNGIGVTQNVLYENQK), LQD (LQDVVNQNAQALNTLVK), LQS (LQSLQTYVTQQLIR). VME1 (membrane protein): EIT (EITVATSR), VAG (VAGDSGFAAYSR), AP3A (protein 3a): WQS (WQLALSK). Retention time in minutes.

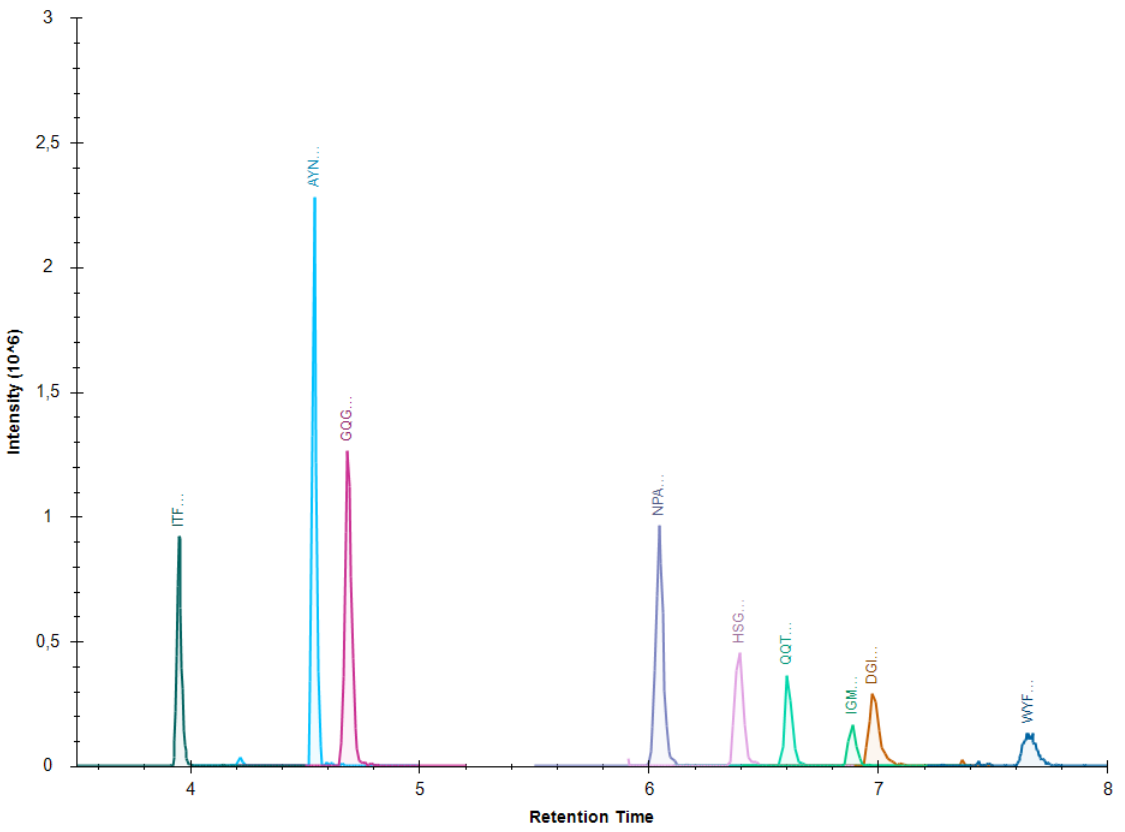

Supplementary figure 2. Nine-min parallel reaction monitoring (PRM) chromatogram of SARS-CoV-2-positive respiratory tract specimen showing nucleoprotein target peptides. The first three residues of each peptide are used to label peptide peaks. NCAP (nucleoprotein): ITF (ITFGGPSDSTGSNQNGER), AYN (AYNVTQAFGR), GQG (GQGVPINTNSSPDDQIGYYR), NPA (NPANNAAIVLQLPQGTTLPK), QQT (QQTVTLLPAADLDDFSK), DGI (DGIIWVATEGALNTPK), IGM (IGMEVTPSGTWLTYTGAIK), WYF (WYFYYLGTGPEAGLPYGANK). ^{15}N-labeled global standard: HSG (HSGFEDELSEVLENQSSQAELK). Retention time in minutes.

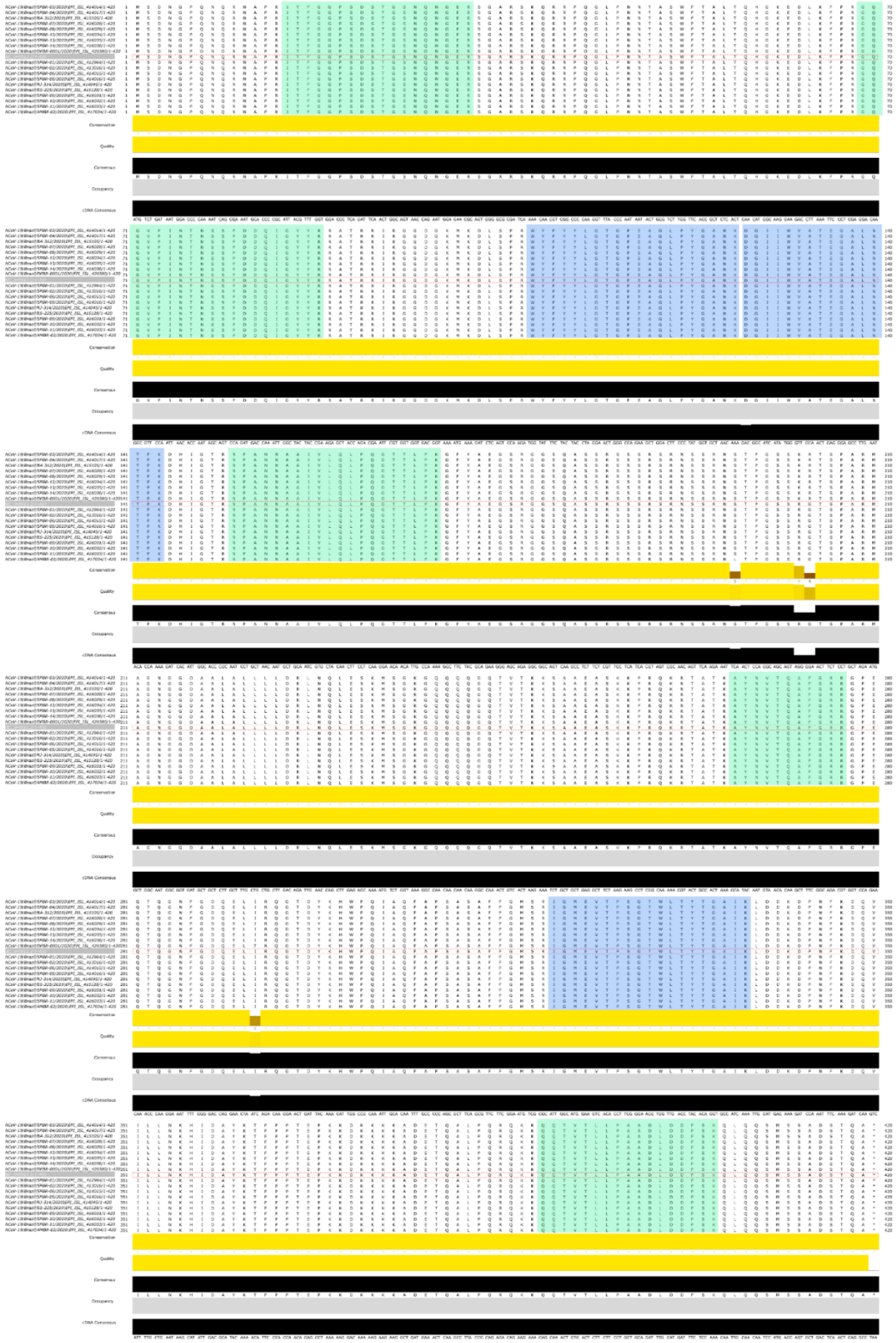

Supplementary figure 3. Multiple sequence alignment (EMBL-EBI Clustal Omega) corresponding to a set of SARS-CoV-2 nucleocapsid phosphoprotein CDSs deposited in GISAID. NCBI reference sequence NC_045512.2 (red dashed box) was included for comparison. Searches were filtered from location and only those sequences with full coverage on this genomic region were included. Segments corresponding to targeted peptides used in the selected reaction monitoring method are shaded in blue and the remaining targeted peptides in the parallel reaction monitoring analysis are shaded in green.

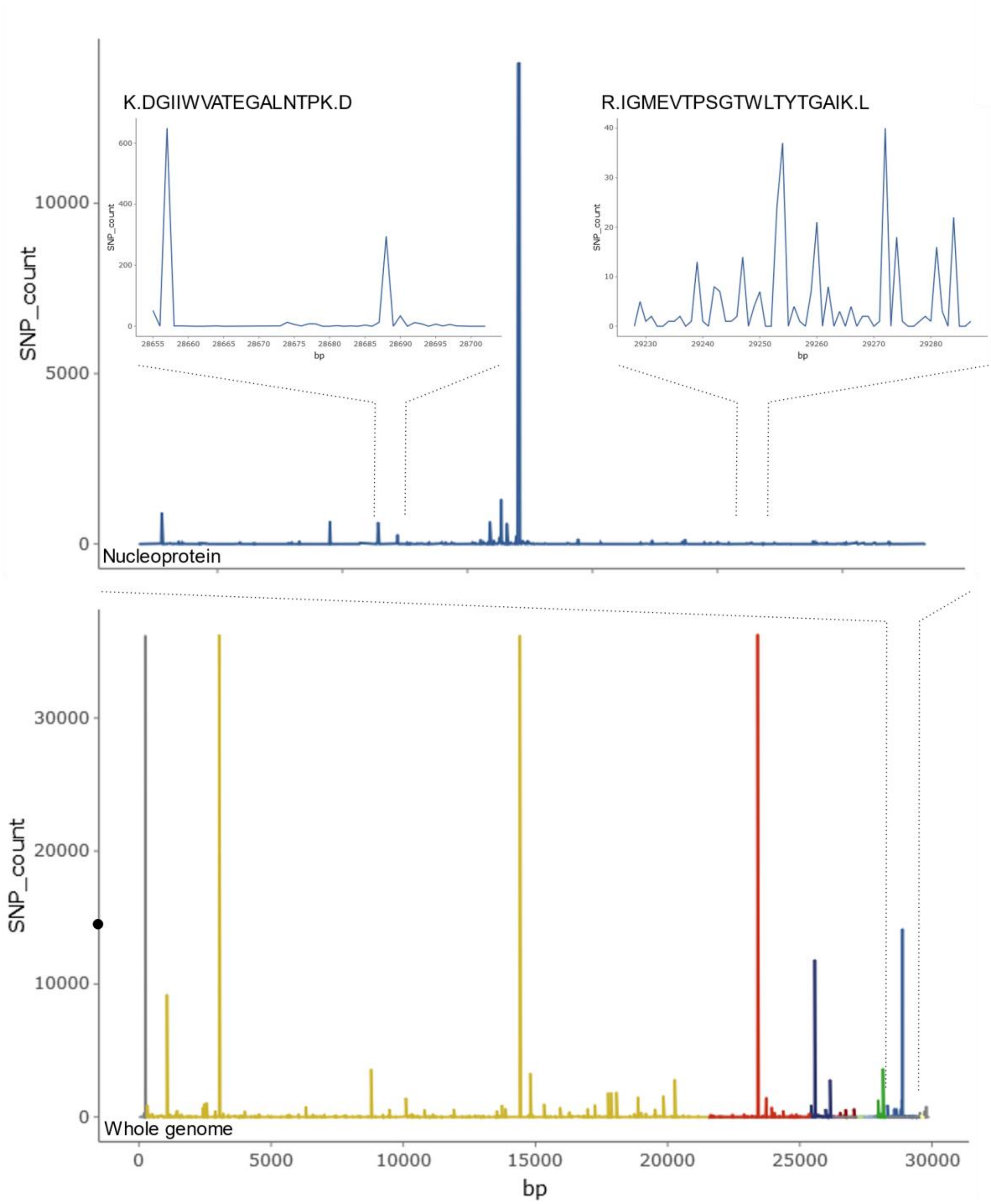

Supplementary figure 4. Analysis of 7666 SARS-CoV-2 genomes using SARS-CoV-2 Alignment Screen [21] depicting the frequencies of single-nucleotide polymorphisms (SNPs). Bottom: Whole SARS-CoV-2 genomes. Top: Expanded nucleoprotein coding region highlighting SNPs frequencies for peptides DGIIWVATEGALNTPK and IGMEVTPSGTWLTYTGAIK. X-axis: base pairs (bp); Y-axis: SNP frequencies.

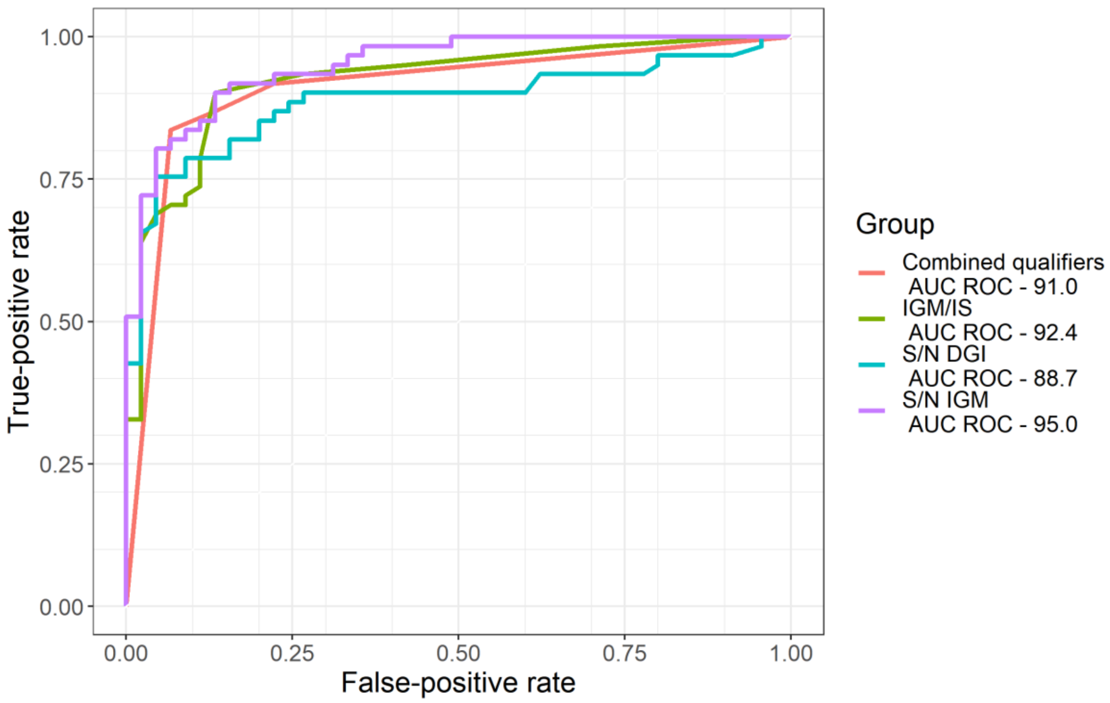

Supplementary figure 5. Receiver operating characteristics (ROC) curves of the qualifiers used for SARS-CoV-2 detection in respiratory tract samples in a testing set (n = 108 biologically independent samples) for Tier 3 assay. AUC ROC: area under the ROC curve; S/N IGM: signal-to-noise for peptide IGMEVTPSGTWLTYTGAIK; S/N DGI: signal-to-noise for peptide DGIIWVATEGALNTPK; and IGM/IS: ratio of peptide IGMEVTPSGTWLTYTGAIK to the surrogate standard. Source data are provided as a Source Data file.

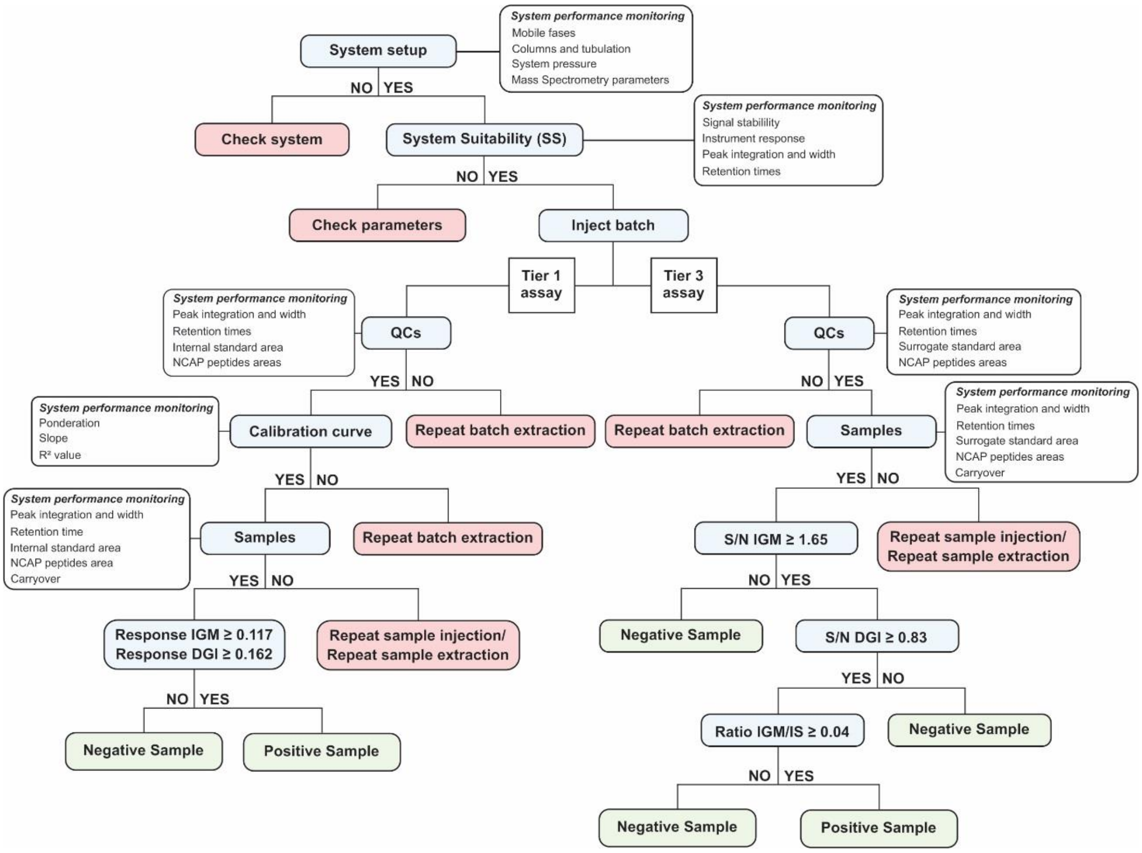

Supplementary figure 6. Entire data processing workflow for sample classification in Tier 1 and Tier 3. QCs: quality control samples; NCAP: nucleoprotein; IGM: IGMEVTPSGTWLTYTGAIK; DGI: DGIIWVATEGALNTPK; S/N: signal-to-noise; and IS: surrogate standard.

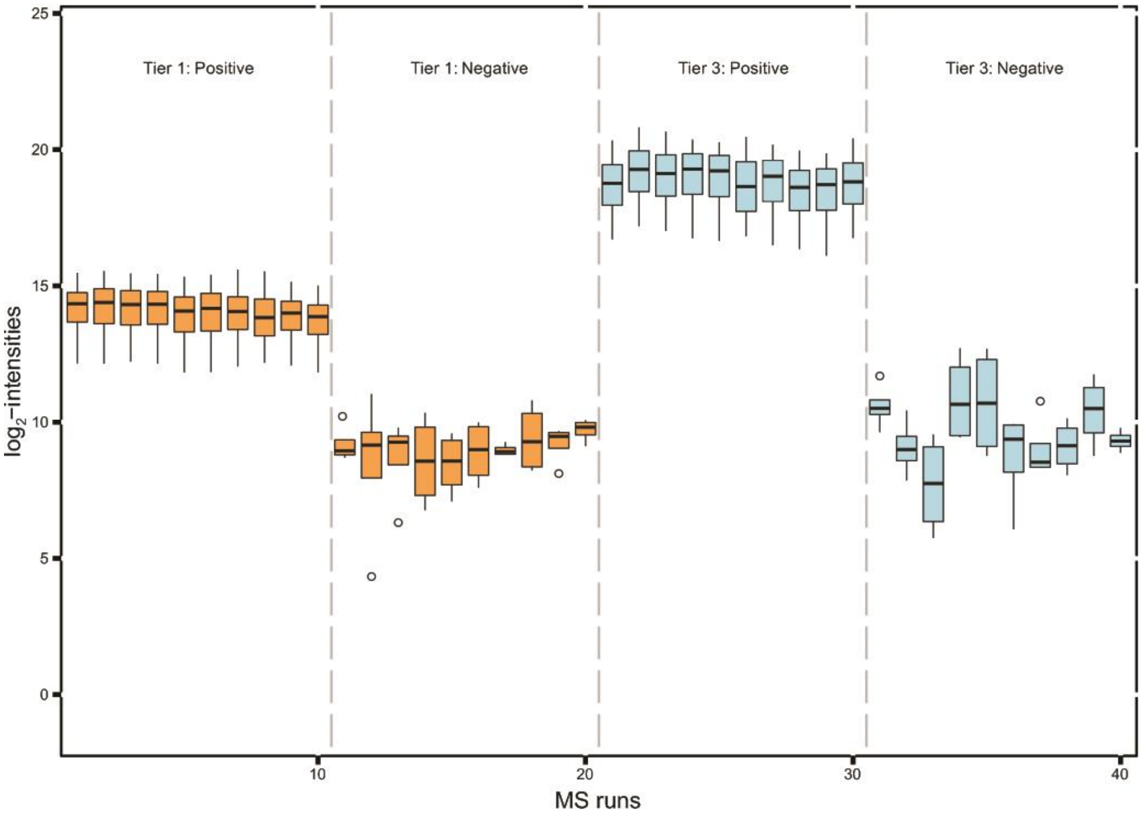

Supplementary figure 7. MSstats quality control (QC) plot of sample reproducibility (n=10 technical replicates) for Tier 1 and Tier 3 assays (independent preparation pool for Tier 1 and 3 assays). X-axis: replicates runs for QC level. Y-axis: normalized intensities (equalize the median peptide) on $\log_2$ scale across all peptides (IGMEVTPSGTWLTYTGAIK and DGIIWVATEGALNTPK) (Tier 1, orange; Tier 3, blue). Boxes indicate the interquartile range (25th percentile and 75th percentile) with the center line indicating the median; minimum whisker, 25th percentile – 1.5 × inter-quantile range (IQR); maximum whisker, 25th percentile + 1.5 × inter-quantile range (IQR); data outside the whiskers are considered outlying points and are plotted individually as a circle. Source data are provided as a Source Data file.

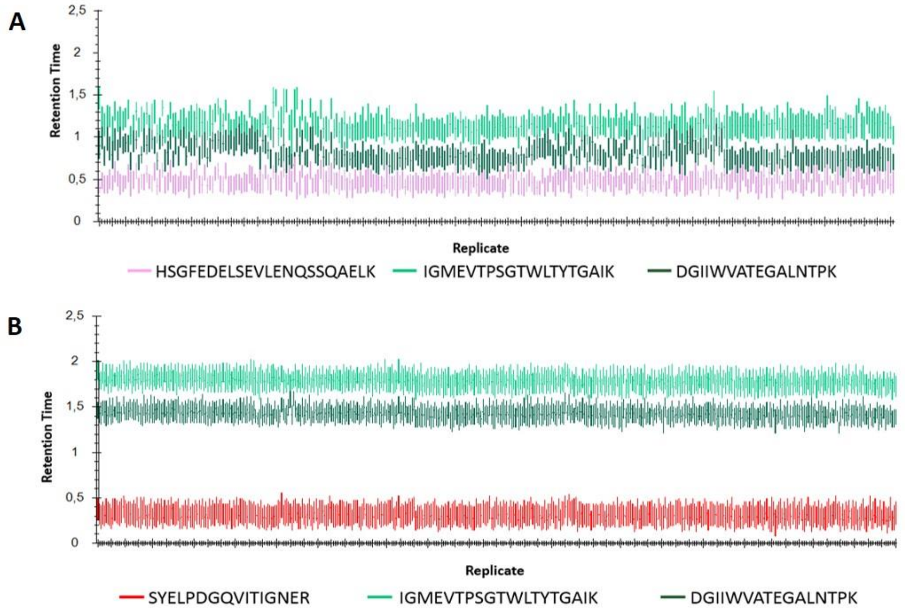

Supplementary figure 8. Reproducibility of the retention time (RT) across five days for (A) Tier 3 and (B) Tier 1 assays. (A) RT= 1.13 ± 0.09 min (CV=8.2%) for IGMEVTPSGTWLTYTGAIK, RT= 0.78 ± 0.11 min (CV= 13.7%) for DGIIWVATEGALNTPK and RT= 0.45 ± 0.05 min (CV=11.8%) for HSGFEDELSEVLENQSSQAELK (surrogate standard Chromogranin A). (B) RT= 1.78 ± 0.06 min (CV=3.0%) for IGMEVTPSGTWLTYTGAIK, RT= 1.39 ± 0.04 min (CV=2.8%) for DGIIWVATEGALNTPK and RT= 0.32 ± 0.03 min (CV=8.6%) for SYELPDGQVITIGNER (human beta actin).

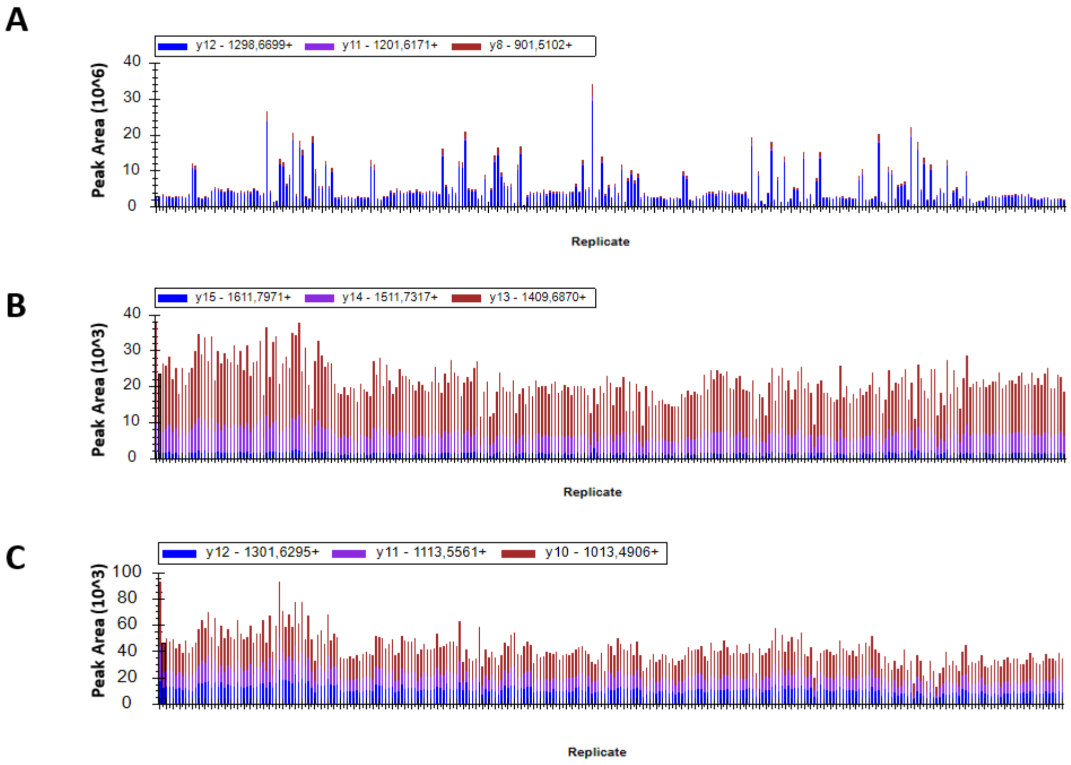

Supplementary figure 9. Reproducibility of the total area across five days of the peak areas for isotope standards and the endogenous beta actin peptides in Tier 1 assay. (A) Beta actin peptide SYELPDGQVITIGNER, (B) ^{15}N-labelled nucleoprotein peptides IGMEVTPSGTWLTYTGAIK and (C) DGIIWVATEGALNTPK.

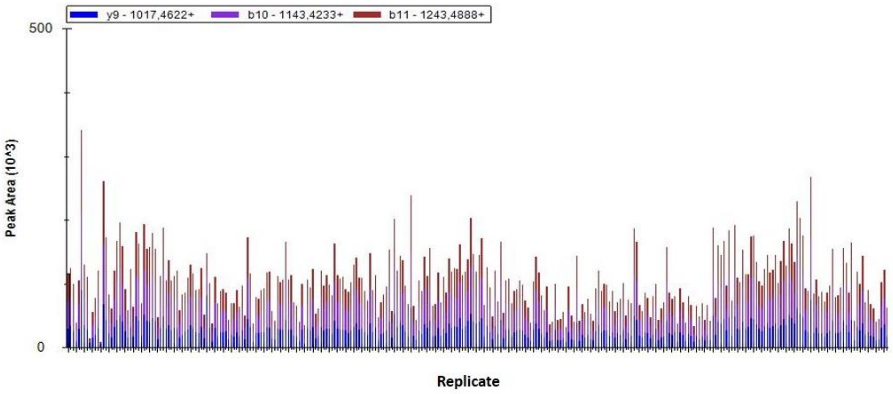

Supplementary figure 10. Reproducibility of the total area across five days of the peptide HSGFEDELSEVLENQSSQAELK (^{15}N-labeled chromogranin A surrogate standard) in the Tier 3 method.

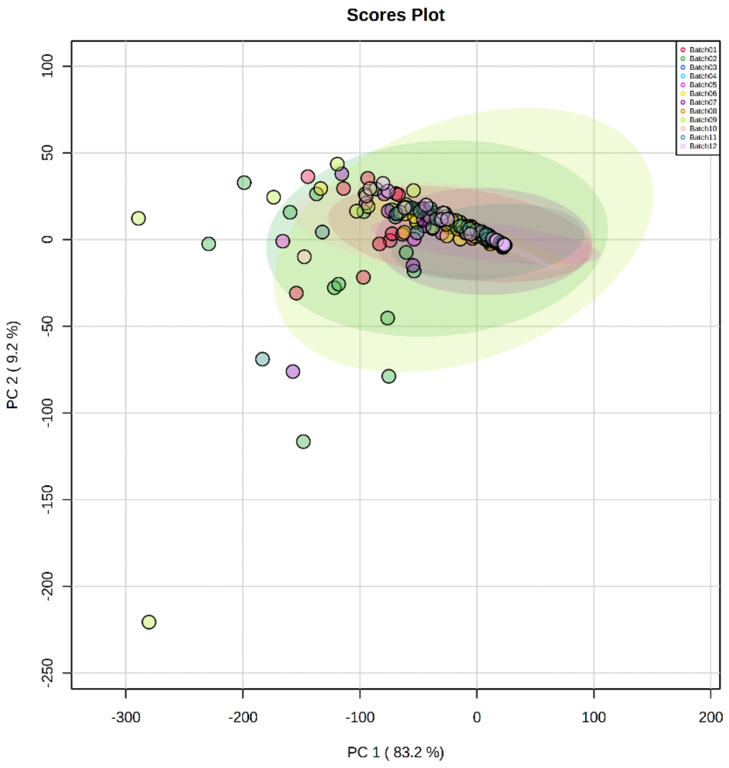

Supplementary figure 11: Score plot of principal component analysis of sample distribution across batches in Tier 3 assay (n = 540 biologically independent samples). Each batch is represented by a color, each dot represents a sample, and circles represent the confidence interval (95%). PC1 (principal component 1) is plotted on x-axis, and PC2 (principal component 2) is plotted on y-axis. Source data are provided as a Source Data file.

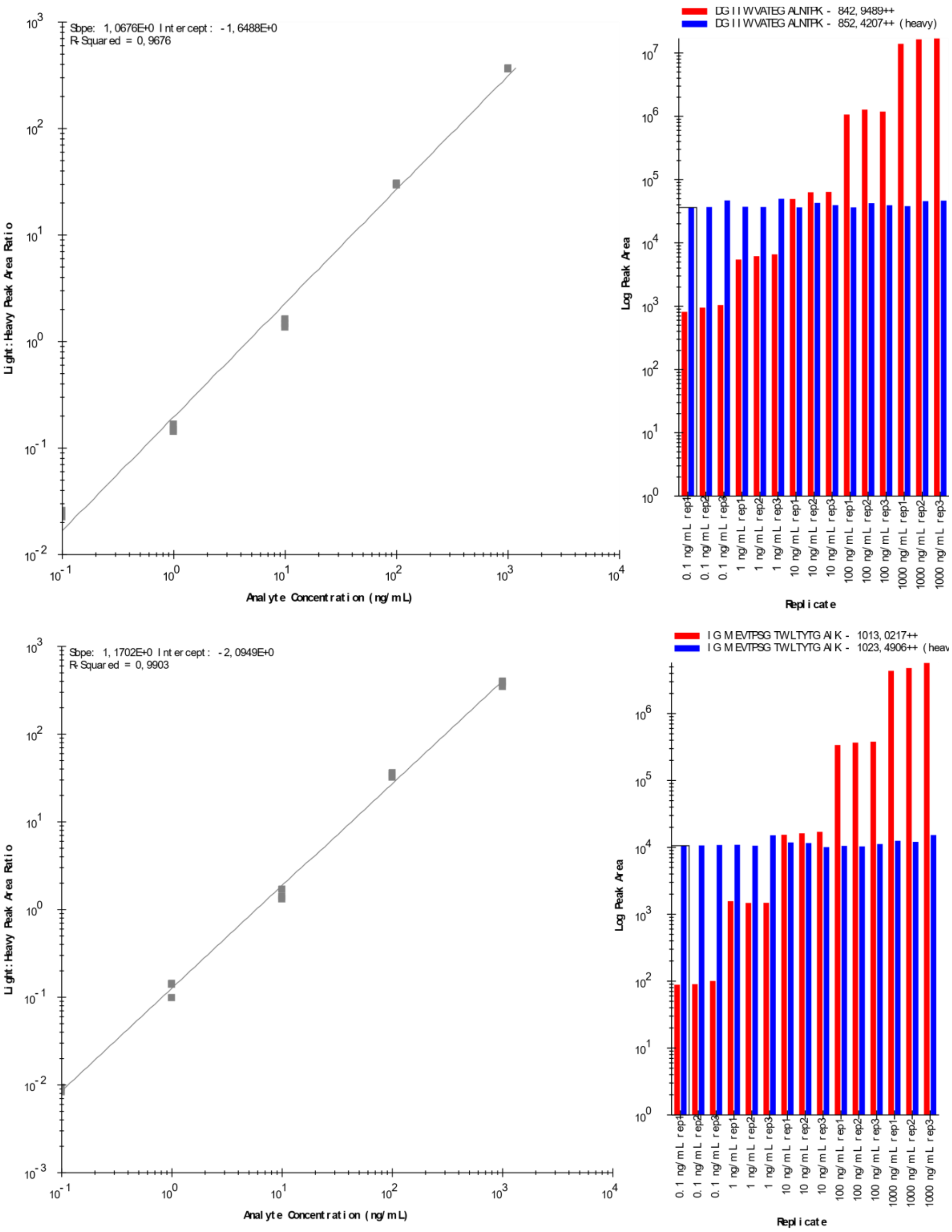

Supplementary figure 12. Linearity of SARS-CoV-2 nucleoprotein in viral transport medium. Nucleoprotein was diluted in the concentration range of 0.1 to 1000 ng/mL and spiked with 10 ng/mL of ^{15}N labeled nucleoprotein. Top: DGIIWVATEGALNTPK. Bottom: IGMEVTPSGTWLTYTGAIK. Left: linear regression from 0.1 to 1000 ng/mL (x-axis) with ratio to heavy as normalization method (y-axis in log scale), three technical replicates per concentration. Right: Peak area replicates comparison bar plot for ^{15}N-labeled nucleoprotein (blue) and nucleoprotein (red).

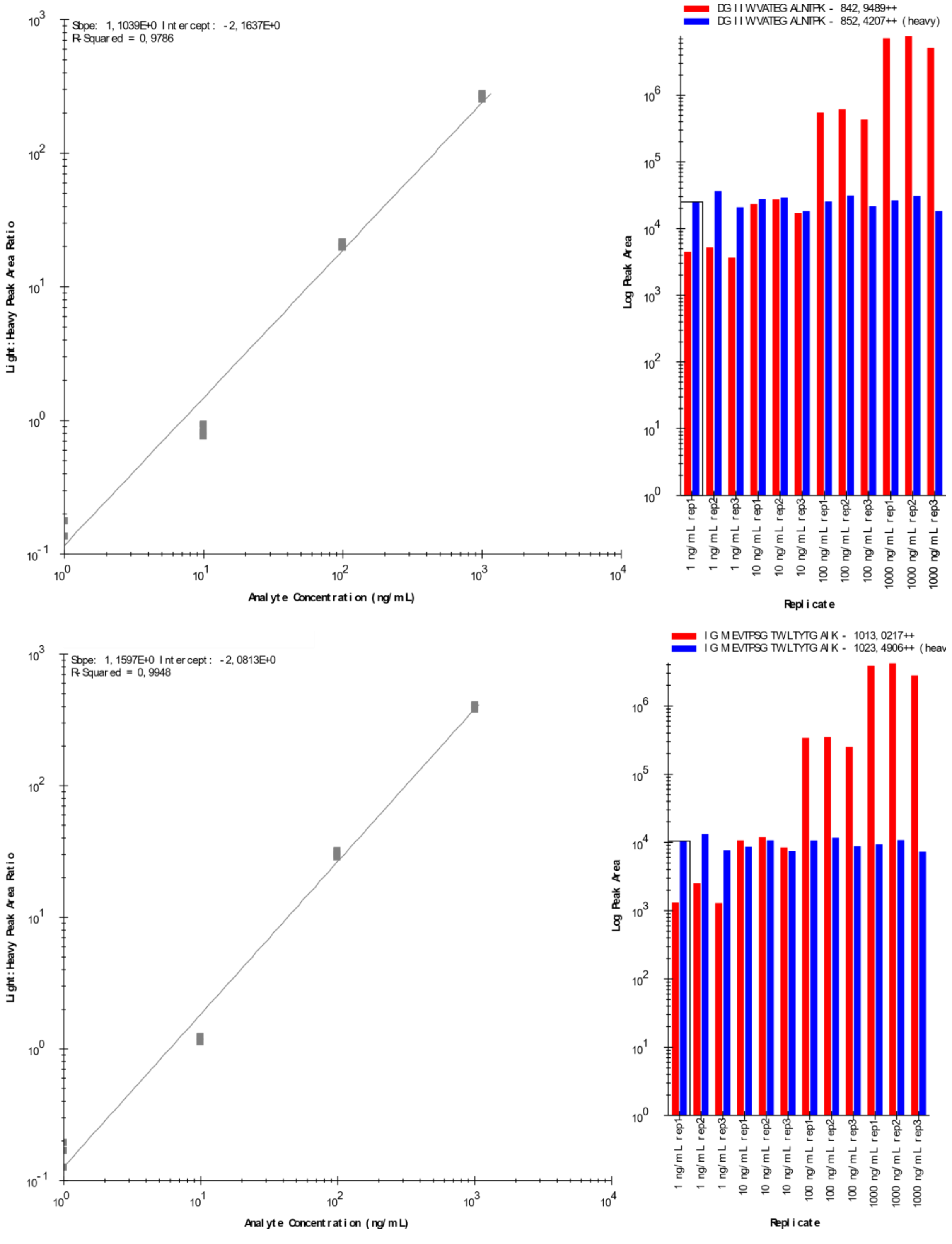

Supplementary figure 13. Linearity of SARS-CoV-2 nucleoprotein in negative pooled samples. Nucleoprotein was diluted in the concentration range of 1 to 1000 ng/mL and spiked with 10 ng/mL of ^{15}N labeled nucleoprotein. Top: DGIIWVATEGALNTPK. Bottom: IGMEVTPSGTWLTYTGAIK. Left: linear regression from 1 to 1000 ng/mL (x-axis) with ratio to heavy as normalization method (y-axis in log scale), three technical replicates per concentration. Right: Peak area replicates comparison bar plot for ^{15}N-labeled nucleoprotein (blue) and nucleoprotein (red).

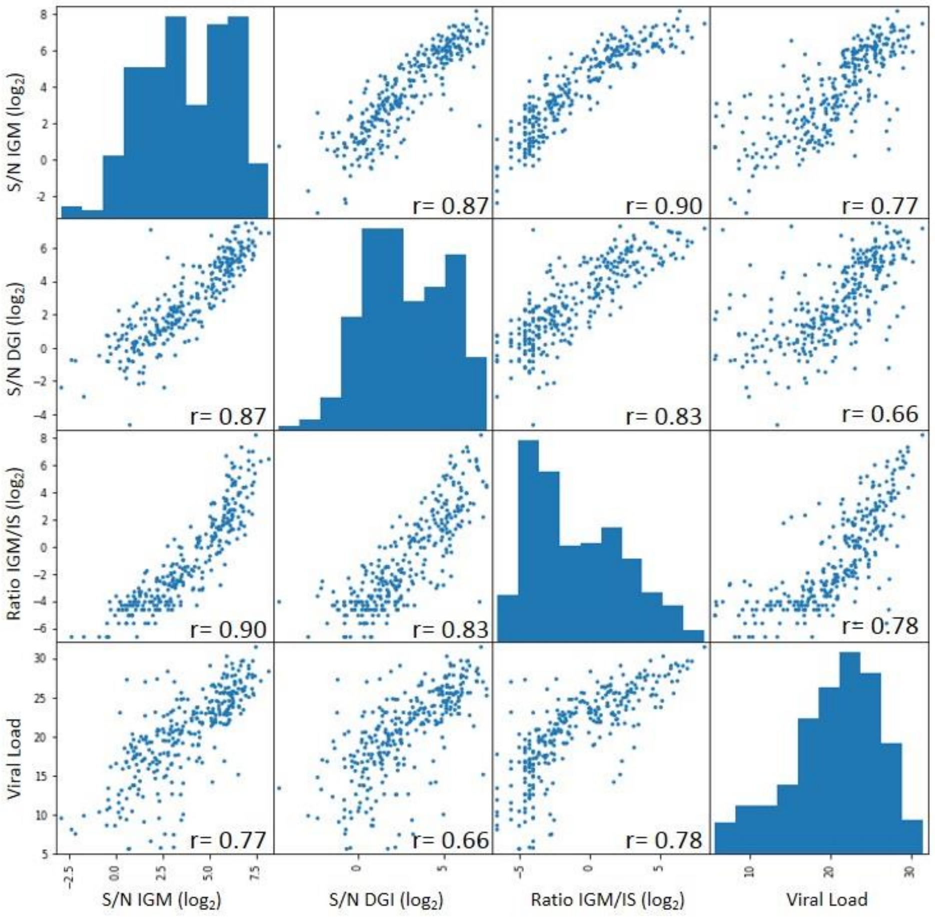

Supplementary figure 14. Scatter plots, histograms, and Pearson's correlation coefficients (r) for 296 positive specimens analyzed by Tier 3 assay and processed in Python/Scikit-learn library [62]. S/N DGI: signal-to-noise for DGIIWVATEGALNTPK; S/N IGM: signal-to-noise for IGMEVTPSGTWLTYTGAIK; Ratio IGM/IS: IGMEVTPSGTWLTYTGAIK normalized by surrogate standard Chromogranin A. VL: viral loads estimated by the equation $y = 3 \times 10^{12} e^{-0.693x}$, where *x* is the cycle threshold value and *y* is the estimated viral load in number of virus copies. All data were $\log_2$ transformed.

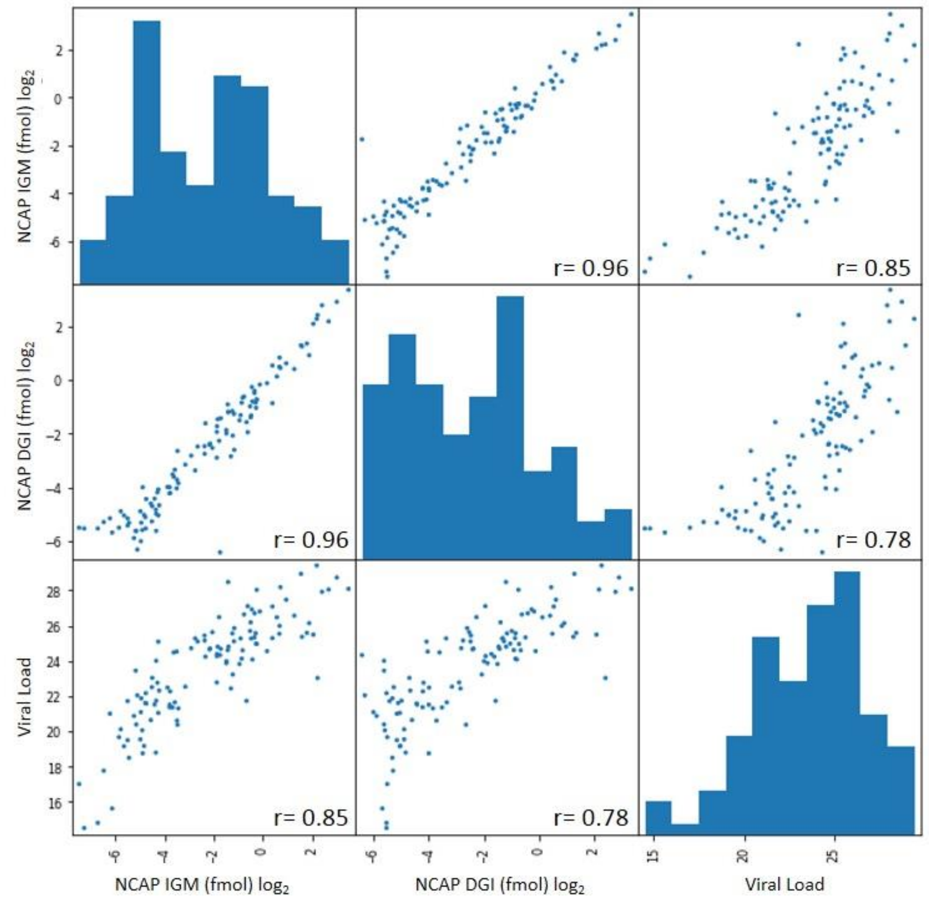

Supplementary figure 15. Scatter plots, histograms, and Pearson's correlation coefficients (r) for 113 positive specimens analyzed by Tier 1 assay and processed in Python/Scikit-learn library [62]. NCAP IGM (fmol): Nucleoprotein in fmol calculated by IGMEVTPSGTWLTYTGAIK; NCAP DGI (fmol): Nucleoprotein in fmol calculated by DGIIWVATEGALNTPK; VL: viral loads estimated by the equation $y = 3 \times 10^{12} e^{-0.693x}$, where *x* is the cycle threshold value and *y* is the estimated viral load in number of virus copies. All data were $\log_2$ transformed.

Supplementary table 1. Amino acid variation retrieved from GISAID hCoV-19 sequences database (last update Aug 11th, 2020) through CoV-GLUE (cov-glue.cvr.gla.ac.uk) for DGIIWVATEGALNTPK and IGMEVTPSGTWLTYTGAIK. Three nucleotides before and after their corresponding coding regions were included to check for modifications in trypsin cleavage sites. Only mutations observed in two or more sequences were included. No insertions or deletions were described within DGIIWVATEGALNTPK and IGMEVTPSGTWLTYTGAIK corresponding coding regions. refNtPosition: reference nucleotide position; refAminoAcid: reference amino acid; repAminoAcid: replaced amino acid; numSeqs: number of sequences with amino acid variation.

	Replacement ID	Replacement	Codon Number	refNtPosition	refAminoAcid	repAminoAcid	numSeqs
DGIIWVATEGALNTPK	N:D:128:Y	D128Y	128	28655	D	Y	60
	N:D:128:H	D128H	128	28655	D	H	2
	N:G:129:D	G129D	129	28658	G	D	2
	N:A:134:V	A134V	134	28673	A	V	11
	N:T:135:I	T135I	135	28676	T	I	7
	N:T:135:P	T135P	135	28676	T	P	3
	N:G:137:V	G137V	137	28682	G	V	7
	N:A:138:S	A138S	138	28685	A	S	3
	N:L:139:F	L139F	139	28688	L	F	29
	N:N:140:T	N140T	140	28691	N	T	13
	N:T:141:I	T141I	141	28694	T	I	5
	N:P:142:S	P142S	142	28697	P	S	11
	N:D:144:Y	D144Y	144	28703	D	Y	27
	N:D:144:N	D144N	144	28703	D	N	7
	N:D:144:H	D144H	144	28703	D	H	4
IGMEVTPSGTWLTYTGAIK	N:R:319:H	R319H	319	29228	R	H	3
	N:I:320:V	I320V	320	29231	I	V	2
	N:G:321:D	G321D	321	29234	G	D	2
	N:M:322:I	M322I	322	29237	M	I	12
	N:M:322:T	M322T	322	29237	M	T	2
	N:E:323:K	E323K	323	29240	E	K	2
	N:V:324:F	V324F	324	29243	V	F	3
	N:V:324:A	V324A	324	29243	V	A	2
	N:T:325:I	T325I	325	29246	T	I	16
	N:T:325:A	T325A	325	29246	T	A	2
	N:P:326:L	P326L	326	29249	P	L	7
	N:P:326:S	P326S	326	29249	P	S	4
	N:S:327:L	S327L	327	29252	S	L	38
	N:G:328:V	G328V	328	29255	G	V	3
	N:G:328:*	G328*	328	29255	G	*	2
	N:T:329:M	T329M	329	29258	T	M	2
	N:W:330:L	W330L	330	29261	W	L	7
	N:L:331:F	L331F	331	29264	L	F	10
	N:T:334:I	T334I	334	29273	T	I	14
	N:A:336:V	A336V	336	29279	A	V	6
	N:A:336:S	A336S	336	29279	A	S	2

.

Supplementary table 2. Efficiency of metabolic ^{15}N-labelling of the recombinant nucleoprotein expressed in *E. coli* verified by digestion of 2 ng of ^{15}N labelled protein followed by analysis by the selected reaction monitoring method. Purity was calculated in triplicate by the summed heavy to light ratios. DGI: DGIIWVATEGALNTPK; IGM: IGMEVTPSGTWLTYTGAIK; Rep.: techinical replicate.

Peptide		Precursor Mz	Precursor Charge	Product Mz	Product Charge	Fragment Ion	Area	Fragment Ion	Area	Fragment Ion	Area	Total area	Ratio %
DGI	Rep. 1	842.95	2	1286.67	1	1286.67	0	1100.59	0	1001.53	33	33	0.08
	Rep. 2	842.95	2	1286.67	1	1286.67	9	1100.59	21	1001.53	323	353	0.63
	Rep. 3	842.95	2	1286.67	1	1286.67	2	1100.59	1	1001.53	34	37	0.12
DGI heavy	Rep. 1	852.42	2	1301.63	1	1301.63	10640	1113.56	11530	1013.49	18881	41051	
	Rep. 2	852.42	2	1301.63	1	1301.63	15176	1113.56	15041	1013.49	25816	56033	**Average= 0.28**
	Rep. 3	852.42	2	1301.63	1	1301.63	7649	1113.56	8127	1013.49	14628	30404	**Purity= 99.72**
IGM	Rep. 1	1013.02	2	1594.85	1	1594.85	0	1495.78	0	1394.73	0	0	0.00
	Rep. 2	1013.02	2	1594.85	1	1594.85	0	1495.78	0	1394.73	20	20	0.12
	Rep. 3	1013.02	2	1594.85	1	1594.85	0	1495.78	0	1394.73	0	0	0.00
IGM heavy	Rep. 1	1023.49	2	1611.80	1	1611.80	781	1511.73	3327	1409.69	9395	13503	
	Rep. 2	1023.49	2	1611.80	1	1611.80	1106	1511.73	4114	1409.69	11641	16861	**Average= 0.04**
	Rep. 3	1023.49	2	1611.80	1	1611.80	581	1511.73	2656	1409.69	6369	9606	**Purity= 99.96**

Supplementary table 3. Tier 3 assay performance based on different qualifiers to discriminate between positive and negative samples.

	Cut-off	Accuracy (95% C.I.)	Sensitivity (95% C.I.)	Specificity (95% C.I.)	AUC (95% C.I.)
S/N IGM	≥1.65	85.8% (77.5% - 94.6%)	91.8% (82.4% - 100.0%)	77.8% (61.5% - 93.8%)	95.0% (89.5% - 99.2%)
S/N DGI	≥0.83	81.1% (71.7% - 90.6%)	90.2% (80.0% - 100.0%)	68.9% (50.0% - 86.7%)	88.7% (79.1% - 96.8%)
IGM/IS	≥0.04	84.9% (75.7% - 94.3%)	93.4% (84.6% - 100.0%)	73.3% (56.2% - 91.7%)	92.4% (85.5% - 98.5%)
Combined qualifiers		87.7% (79.4% - 95.2%)	83.6% (70.6% - 95.7%)	93.3% (82.4% - 100.0%)	91.0% (83.6% - 98.1%)

AUC: area under the curve; CI: confidence interval; S/N IGM: signal-to-noise for peptide IGMEVTPSGTWLTYTGAIK; S/N DGI: signal-to-noise for peptide DGIIWVATEGALNTPK; and IGM/IS: ratio of peptide IGMEVTPSGTWLTYTGAIK to the surrogate standard. Source data are provided as a Source Data file.

Supplementary table 4. Tier 1 assay performance based on limits of detection to discriminate between positive and negative samples.

	Cut-off	Accuracy (95% CI)	Sensitivity (95% CI)	Specificity (95% CI)
LoD IGM	≥0.117	83.4% (79.6% - 86.5%)	89.1% (84.4% - 92.5%)	77.3% (71.3% - 82.4%)
LoD DGI	≥0.162	81.1% (77.2% - 84.5%)	79.5% (73.8% - 84.2%)	82.9% (77.3% - 87.3%)
Combined LoDs		87.2% (83.8%-90.0%)	78.2% (72.4% - 83.0%)	96.8% (93.5% - 98.4%)

LoD IGM: limit of detection for IGMEVTPSGTWLTYTGAIK; LoD DGI: limit of detection for DGIIWVATEGALNTPK; CI: confidence interval. Source data are provided as a Source Data file.

Supplementary table 5. Lack of interference in respiratory samples from patients with infections other than SARS-CoV-2 infections.

Sample	Interferent	SARS-CoV-2 presence
1	Rhinovirus/Enterovirus	Undetected
2	Rhinovirus/Enterovirus/Human metapneumovirus	Undetected
3	Rhinovirus/Enterovirus	Undetected
4	Coronavirus 229E	Undetected
5	Rhinovirus/Enterovirus	Undetected
6	Respiratory syncytial virus	Undetected
7	Rhinovirus/Enterovirus	Undetected
8	Coronavirus HKU1/Rhinovirus/Enterovirus	Undetected
9	Parainfluenza 4	Undetected
10	Influenza A/H1-2009	Undetected
11	Influenza A/H1-2009	Undetected
12	Parainfluenza 1	Undetected
13	Rhinovirus/Enterovirus/Human metapneumovirus	Undetected
14	Coronavirus NL63	Undetected
15	Human metapneumovirus	Undetected
16	Rhinovirus/Enterovirus	Undetected
17	Rhinovirus/Enterovirus	Undetected
18	Rhinovirus/Enterovirus	Undetected
19	Rhinovirus/Enterovirus	Undetected
20	Rhinovirus/Enterovirus	Undetected
21	Rhinovirus/Enterovirus	Undetected
22	Rhinovirus/Enterovirus	Undetected
23	Rhinovirus/Enterovirus	Undetected
24	Rhinovirus/Enterovirus	Undetected
25	Rhinovirus/Enterovirus	Undetected
26	Rhinovirus/Enterovirus	Undetected
