# Supplementary material for: Establishing a mass spectrometry-based system for rapid detection of SARS-CoV-2 in large clinical sample cohorts
Source: Nat Commun. 2020 Dec 3;11:6201. doi: 10.1038/s41467-020-19925-0 (PMC7713649; doi:10.1038/s41467-020-19925-0)
Supplement: Supplementary file 3 — Reporting Summary [file 41467_2020_19925_MOESM3_ESM.pdf]

nature research

Corresponding author(s): Valdemir Melechco Carvalho

Last updated by author(s): Oct 29, 2020

Reporting Summary

Nature Research wishes to improve the reproducibility of the work that we publish. This form provides structure for consistency and transparency in reporting. For further information on Nature Research policies, see our Editorial Policies and the Editorial Policy Checklist.

Statistics

For all statistical analyses, confirm that the following items are present in the figure legend, table legend, main text, or Methods section.

n/a	Confirmed	
☐	☒	The exact sample size (n) for each experimental group/condition, given as a discrete number and unit of measurement
☐	☒	A statement on whether measurements were taken from distinct samples or whether the same sample was measured repeatedly
☐	☒	The statistical test(s) used AND whether they are one- or two-sided<br>*Only common tests should be described solely by name; describe more complex techniques in the Methods section.*
☒	☐	A description of all covariates tested
☒	☐	A description of any assumptions or corrections, such as tests of normality and adjustment for multiple comparisons
☐	☒	A full description of the statistical parameters including central tendency (e.g. means) or other basic estimates (e.g. regression coefficient) AND variation (e.g. standard deviation) or associated estimates of uncertainty (e.g. confidence intervals)
☐	☒	For null hypothesis testing, the test statistic (e.g. F, t, r) with confidence intervals, effect sizes, degrees of freedom and P value noted<br>*Give P values as exact values whenever suitable.*
☒	☐	For Bayesian analysis, information on the choice of priors and Markov chain Monte Carlo settings
☒	☐	For hierarchical and complex designs, identification of the appropriate level for tests and full reporting of outcomes
☒	☐	Estimates of effect sizes (e.g. Cohen's d, Pearson's r), indicating how they were calculated

Our web collection on statistics for biologists contains articles on many of the points above.

Software and code

Policy information about availability of computer code

Data collection	Thermo Scientific XCalibur (version 4.2.47), TraceFinder (version 4.1) and Aria MX (version 2.5) were used to acquire mass spectrometric data.
Data analysis	MaxQuant (version 1.6.14) was used to process untargeted raw data. Skyline (daily version 20.1.9.234) was used to build the spectral library, and to select transitions for PRM and SRM assays. AutoQC Loader (version 1.1.0.18345) and Panorama (version 18.2) were used in quality control. CoV-GLUE was used to verify amino acid variation including substitutions, insertions and deletions. Clustal Omega server and JalView (2.11.0) alignment editor were used to verify genome alignment. Skyline was also used to import all the targeted acquisitions and system suitability tests and perform peak detection, peak integration and background noise calculation (Tier 3) as soon as peptide quantification (Tier 1). Excel (for Office 365) was used to consolidate data processed by Skyline and also calculate signal to background and signal to internal standard ratios, limits of assays (LoB and LoD), sample stability and carryover. MSstats (plugin for Skyline) was used to evaluate quality control reproducibility of both Tiers. Python/Scikit-learn library was used to evaluate correlations between targeted proteomics and real-time RT-PCR data. EP evaluator (version 12) was used to determine method sensitivity and specificity, reproducibility variation and limit of quantification. R packages (version 3.6.0; packages ggplot2, pROC) were used to analyze batch effects and determine the diagnostic accuracy for combination of parameters to Tier 1 and Tier 3 assays.

For manuscripts utilizing custom algorithms or software that are central to the research but not yet described in published literature, software must be made available to editors and reviewers. We strongly encourage code deposition in a community repository (e.g. GitHub). See the Nature Research guidelines for submitting code & software for further information.

Data

Policy information about availability of data

All manuscripts must include a data availability statement. This statement should provide the following information, where applicable:

- Accession codes, unique identifiers, or web links for publicly available datasets
- A list of figures that have associated raw data
- A description of any restrictions on data availability

The mass spectrometry untargeted proteomics data (raw files and spectral library) have been deposited to the ProteomeXchange Consortium via the PRIDE 61 partner repository with the dataset identifier PXD021328 (https://www.ebi.ac.uk/pride/archive/projects/PXD021328). Targeted analyses proteomics data (PRM 60- and 9-min analyses, SRM analyses and summary datasheet) are available through the Panorama repository 52 with the dataset identifier PXD019300 (https://panoramaweb.org/labkey/Fleury_SARS-Cov-2.url). SARS-CoV-2 protein sequence information used in this study are available from UniProt (https://covid-19.uniprot.org). Multiple sequence alignments analysis of SARS-CoV-2 genomes (as of April 14, 2020 and August 11, 2020) are available from GISAID through CoV-GLUE (https://cov-glue.cvr.gla.ac.uk). Reference genome sequence used in this study was from National Center for Biotechnology Information (NCBI) with GenBank accession NC_045512.2 (https://www.ncbi.nlm.nih.gov/nuccore/NC_045512). All other data generated are included in figures and tables in this published article. Source data are provided with this paper.

Field-specific reporting

Please select the one below that is the best fit for your research. If you are not sure, read the appropriate sections before making your selection.

- [x] Life sciences
- [ ] Behavioural & social sciences
- [ ] Ecological, evolutionary & environmental sciences

For a reference copy of the document with all sections, see nature.com/documents/nr-reporting-summary-flat.pdf

Life sciences study design

All studies must disclose on these points even when the disclosure is negative.

Sample size	The sample size was determined according to the Clinical and Laboratory Standards Institute Guideline (CLSI) guideline for evaluation of the qualitative method - EP 12 (Garrett, P. E., Lasky, F. D., Meier, K. L., Clark, L. W. & Clinical and Laboratory Standards Institute. User protocol for evaluation of qualitative test performance : approved guideline. 2nd edn, 2008), that recommend as minimum 100 samples (50 positives and 50 negatives). The total number of specimens analyzed for comparative studies was 540 for Tier 3 (311 positives and 229 negatives by real time RT–PCR) and 445 (229 positives and 216 negatives by real time RT–PCR).
Data exclusions	Data were excluded from method validation (accuracy, limits of quantification, limits of detection and precision) when they did not meet requirements established during validation such as the presence of carryover and minimum surrogate standard area (Tier 3) or the presence of carryover and minimum internal standard or beta actin area (Tier 1).
Replication	Reproducibility was evaluated using negative and positive pooled samples over ten days and two replicates per day. Each Tier assay evaluated a different pooled of positive samples. The statistical analysis was performed using MSstats plugin for Skyline (Choi, M. et al. MSstats: an R package for statistical analysis of quantitative mass spectrometry-based proteomic experiments. Bioinformatics 30, 2524-2526 (2014)) and EP Evaluator (version 12). All replication experiments were succesfull and presented coefficient of variation less than 20%.
Randomization	Samples previously defined as positive or negative for SARS-CoV-2 by real time RT-PCR were randomly allocated in the robotic liquid handler for analysis.
Blinding	Blinding was not performed in this study since RT-PCR results were used to allow methods comparison.

Reporting for specific materials, systems and methods

We require information from authors about some types of materials, experimental systems and methods used in many studies. Here, indicate whether each material, system or method listed is relevant to your study. If you are not sure if a list item applies to your research, read the appropriate section before selecting a response.

Materials & experimental systems

n/a	Involved in the study	
x		Antibodies
x		Eukaryotic cell lines
x		Palaeontology and archaeology
x		Animals and other organisms
	x	Human research participants
x		Clinical data
x		Dual use research of concern

Methods

n/a	Involved in the study	
x		ChIP-seq
x		Flow cytometry
x		MRI-based neuroimaging

Human research participants

Policy information about studies involving human research participants

Population characteristics	This study used exclusively unidentified specimens previously analyzed by real-time RT-PCR test for SARS-CoV-2. No clinical or demographic information was obtained.
Recruitment	Samples were collected from hospitalized patients in São Paulo (Brazil) with clinical symptoms for COVID-19.
Ethics oversight	Approved by the Instituto Fleury Ethics Committee and registered at Plataforma Brasil (certificate number: 31686420.6.0000.5474).

Note that full information on the approval of the study protocol must also be provided in the manuscript.
